# Point-of-care ultrasound of the common carotid arteries for detection of large vessel occlusion stroke: Results of the POCUS-LVO study

**DOI:** 10.1177/23969873251315337

**Published:** 2025-01-30

**Authors:** João Pinho, Anna Tyurina, Celina Hartmann, Omar Abu Audeh, Pardes Habib, Ramy Abdelnaby, Oliver Matz, Marc Felzen, Jörg C. Brokmann, Martin Wiesmann, Jörg B. Schulz, Omid Nikoubashman, Arno Reich

**Affiliations:** 1 Department of Neurology, University Hospital RWTH Aachen, Aachen, Germany; 2 Department of Neurosurgery, School of Medicine, Stanford University, Stanford, USA; 3 Department of Anesthesiology, University Hospital RWTH Aachen, Aachen, Germany; 4 Emergency Department, University Hospital RWTH Aachen, Aachen, Germany; 5 Department of Diagnostic and Interventional Neuroradiology, University Hospital RWTH Aachen, Aachen, Germany; 6 JARA-BRAIN Institute Molecular Neuroscience and Neuroimaging, Forschungszentrum Jülich GmbH and RWTH Aachen University, Aachen, Germany

**Keywords:** Stroke, large vessel occlusion, mechanical thrombectomy, ultrasound, sensitivity and specificity

## Abstract

**Introduction:**

Distal arterial occlusions can cause measurable changes in the flow wave profile in proximal segments of the feeding artery. Our objective was to study the diagnostic accuracy of point-of-care ultrasound (POCUS) of the common carotid arteries (CCA) for detection of anterior circulation large vessel occlusion (ac-LVO) in patients with suspected stroke.

**Patients and methods:**

We conducted a prospective, single-center, observational study of adult patients with suspected stroke admitted in the emergency department. Flow wave profiles of both CCAs were generated by non-specialists using POCUS as soon as possible after admission. ac-LVO was defined as an internal carotid artery or M1 occlusion in CT- or MR-angiography. The diagnostic performances for detection of ac-LVO using flow wave parameters were calculated.

**Results:**

Among 283 patients recruited during a 10-month period, 257 patients (91%) had CCA ultrasound images of sufficient quality and were included for analysis. The mean age was 75 years (IQR 62–83), 131 were female (51.0%), median baseline NIHSS was 2 (IQR 0–5). The most frequent final diagnosis was ischemic stroke (49.4%), ac-LVO was present in 30 patients (11.9%). The median duration of POCUS was 3 min (IQR 2–5). Among all flow wave parameters, the highest diagnostic accuracy for ac-LVO detection was found for end-diastolic velocity difference between sides (AUC = 0.90, 95%CI = 0.85–0.93), with a specificity of 83% (95%CI = 78–88%) at a predefined sensitivity threshold of 80%.

**Discussion and conclusion:**

POCUS of the CCA in patients with suspected stroke can predict the presence of ac-LVO. These results need to be replicated in a prehospital setting.

## Graphical abstract

**Graphical abstract:**
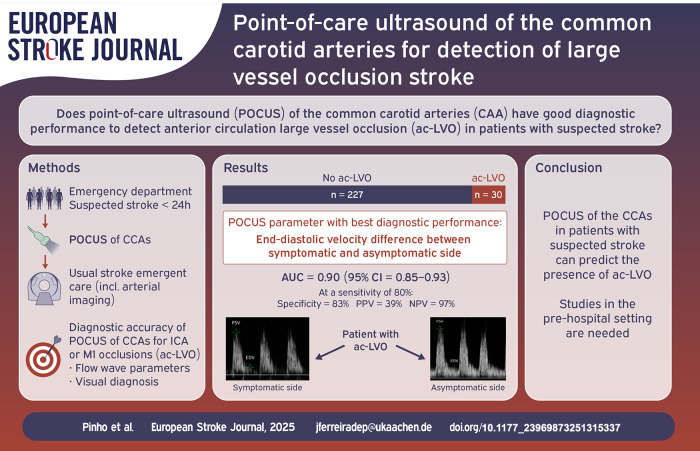

## Introduction

Mechanical thrombectomy (MT) with or without intravenous thrombolysis is currently the best available acute revascularization treatment for patients with acute ischemic stroke (AIS) caused by anterior circulation large vessel occlusion (ac-LVO).^1,2^ One of the most important factors contributing to the benefit of acute revascularization treatments in stroke patients is a short time interval between symptom onset and revascularization.^3,4^ One of the major challenges of stroke medicine is to increase public awareness of stroke as a treatable emergent condition, and to organize prehospital and hospital resources to provide the most adequate acute treatment to the right patient as quickly as possible.^5^ MT, which is typically available in high-volume hospitals, requires highly trained interventionalists with expertise, dedicated multidisciplinary stroke teams and access to complex diagnostic and treatment resources.^5,6^ Non-MT capable centers need to transfer patients for endovascular treatment, which is usually associated with delay and decrease in the number of patients eligible to treatment.^7^ An alternative strategy is to accurately identify patients with large vessel occlusion early in the prehospital phase and to transfer these patients directly to a MT-capable center. There are several screening tools, namely clinical stroke scales, CT-angiography on mobile stroke units, transcranial ultrasonography, and electroencephalography,^8^ which have been studied for this use. Some of these tools have important limitations in respect to large scale implementation, costs and suboptimal screening performance.^9^ We demonstrated, in a small proof-of-concept study, that changes in flow wave profiles of the common carotid arteries (CCA) could be indicative of an ac-LVO in acute stroke patients.^10^

The aim of this study was to analyze the diagnostic accuracy of point-of-care ultrasound (POCUS) of the CCA for diagnosis of ac-LVO in a population of patients with suspected stroke.

## Patients and methods

The POCUS-LVO study was a single-center, prospective, investigator-initiated, observational study, which consisted on three phases.

### Training phase

Between November-December 2022, three fourth-year medical students received two 1-h ultrasound workshops with experienced neurologists. Following these workshops, each medical student regularly visited the neurology wards to perform unsupervised duplex ultrasound of both CCAs in 50 unselected patients. Images were stored and retrospectively evaluated by two senior neurologists certified by the German Society for Ultrasound in Medicine (DEGUM), who were blinded for the examiner and the time order in which each examination was performed. Total duration and the feasibility of the examination (generation of images of sufficient quality to correctly evaluate the flow wave parameters) were collected. The device used for the study was a portable multipurpose POCUS device (C3 curved array scanner HD, Clarius^®^, UK) connected to a handheld tablet computer (iPad mini, Apple^®^, California, USA) for visualization.

### Patient recruitment phase

Patient recruitment was conducted in the ED of a university hospital between March/2023 and January/2024. Adult patients admitted to the neurological ED with suspected stroke or transient ischemic attack (TIA) in the previous 24 h were included as soon as possible after admission and before any revascularization therapy. The exclusion criteria were: absent written informed consent, pregnancy, suspected or confirmed cervical trauma, cervical wounds or active inflammatory cervical lesions, cervical surgery in the previous 30 days, any contraindication for cervical rotation and predictable delay of the routine care induced by the performance of the POCUS. The medical students performed the POCUS examinations and were organized in shifts in the ED covering an average of 32 hours/workweek mainly during regular work hours (8:00am–04:00pm). They were instructed to perform POCUS examinations of both CCAs as fast as possible, to visualize the middle segment of CCA on both sides, to generate flow wave profiles of both vessels using duplex imaging, and to measure the peak systolic velocities (PSV) and end-diastolic velocities (EDV) with angle correction <60° and parallel to the vessel wall. Measurements of PSV and EDV were performed in a single randomly selected heart cycle. Resistance index (RI) and pulsatility index (PI) were calculated. The treating team was encouraged to simultaneously carry on standard patient evaluation. Medical students were instructed to interrupt the POCUS examination if it could interfere or delay routine care of the participants. The images were uploaded in the local imaging archive and were not evaluated at this point. Routine monthly assessments of treatment time metrics performed by workers of the Department of Neurology and Department of Diagnostic and Interventional Neuroradiology not primarily involved in the study did not raise any concerns concerning delays of acute stroke treatment during the study period.

Demographic and clinical information of participants was collected prospectively and included vascular risk factors, comorbidities, admission blood pressure values, and baseline National Institutes of Health Stroke Scale (NIHSS). Presence of ac-LVO was defined as occlusion of the cervical and/or intracranial segments of the internal carotid artery and/or occlusion of the proximal segment of the middle cerebral artery (M1) in the initial emergent imaging (CT-angiography or MR-angiography), as evaluated by the neuroradiologist on call. Presence of moderate-to-severe internal carotid artery stenoses (50%–99%) was collected from clinical records based on the reports of CT-angiography, MR-angiography or ultrasound performed as part of the routine care of these patients. Final diagnosis was collected retrospectively from the hospital discharge letter.

### Imaging visual assessment phase

**Figure 1.**
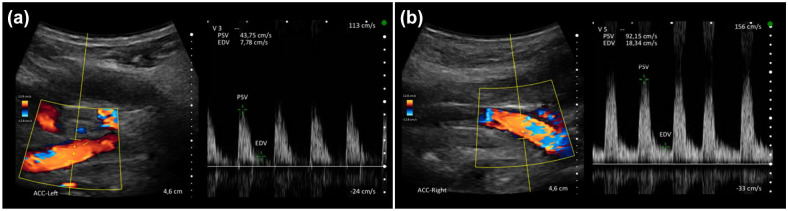
Example of flow wave patterns of the common carotid arteries in a patient with an acute occlusion of the left carotid-T, showing reduced peak-systolic velocities, reduced end-diastolic velocities and increased pulsatility (A) in comparison to the right side (B).

After the recruitment phase, POCUS images were anonymized for independent rating by two DEGUM-certified neurologists. Patients with unavailable POCUS images for at least one side or with POCUS images of insufficient quality were excluded from the study. For each case, images of CCAs were placed side by side and the symptomatic side was provided. The symptomatic side was defined as the side contralateral to the focal neurological deficits in patients with ischemic stroke, TIA, intracranial hemorrhage, focal seizures, migraine with aura or psychogenic focal neurological deficits, or predefined as the right side in patients with other stroke mimics with no clear focal deficits or for patients in whom the symptomatic side was impossible to determine. The two raters identified CCA flow wave patterns compatible with ac-LVO (visual diagnosis) fulfilling the following criteria: ipsilateral increased pulsatility, ipsilateral absent or greatly decreased EDV, relevant asymmetry in side comparison (Figure 1).^10^ Disagreements were settled by a third DEGUM-certified neurologist.

### Statistical analysis

POCUS feasibility and POCUS duration throughout the training phase was compared by grouping the exams in tertiles according to the order they were performed by each student and performing a chi-square test and a Kruskal–Wallis test, respectively.

We estimated the size of the study population based on an expected specificity of 80% and an ac-LVO prevalence of 10%.^11^ Assuming a maximal marginal error of 5%, we set the study population size at 273 patients.^12^ We compared the baseline clinical characteristics of patients with and without ac-LVO by using chi-square tests and Mann–Whitney tests as appropriate. Results are presented as number (%) or median (interquartile range (IQR)). The diagnostic performance of the following parameters for the detection of ac-LVO were calculated: PSV (symptomatic CCA), EDV (symptomatic CCA), RI (symptomatic CCA), PI (symptomatic CCA), PSV difference between sides (symptomatic CCA–asymptomatic CCA), EDV difference between sides (symptomatic CCA–asymptomatic CCA), RI difference between sides (symptomatic CCA–asymptomatic CCA), PI difference between sides (symptomatic CCA–asymptomatic CCA), visual diagnosis. Diagnostic accuracy for each of the parameters was assessed by calculating the AUC and respective 95% confidence interval (95%CI). We performed analyses of the diagnostic performances for the continuous POCUS variables by setting a threshold for sensitivity at 80%, choosing the associated parameter value with the highest specificity and calculating the positive predictive value (PPV), negative predictive value (NPV) and the positive likelihood ratio (PLR) and their respective 95%CI. We compared the receiver operating characteristic (ROC) curves of the three POCUS parameters with the highest AUC with the ROC curve of the three POCUS parameters with the lowest AUC using the DeLong test for pairwise comparisons. Interobserver agreement for visual diagnosis of ac-LVO in POCUS was evaluated using Cohen’s kappa (κ).

As sensitivity analyses, we calculated the diagnostic performance of the POCUS parameters for detection of occlusions of ICA, M1, M2 and basilar artery, and by calculating the diagnostic performance of POCUS only in ischemic stroke patients. To assess a possible confounding effect induced by the presence of a 50%–99% internal carotid artery stenosis ipsilateral to suspected ischemic stroke, we compared its frequency between the groups with false positive and true negative findings according the best performing POCUS criteria.

The statistical analyses were performed in SPSS^®^ Statistics (version 28.0.1.0, IBM Corporation, Armonk, NY) and MedCalc^®^ Statistical Software (version 20.111, Ostend, Belgium). Threshold for statistical significance was set at an alpha value of 0.05. No missing data imputation was performed. This report follows the Standards for Reporting of Diagnostic Accuracy recommendations (Supplemental Table 1).^13^

## Results

**Figure 2.**
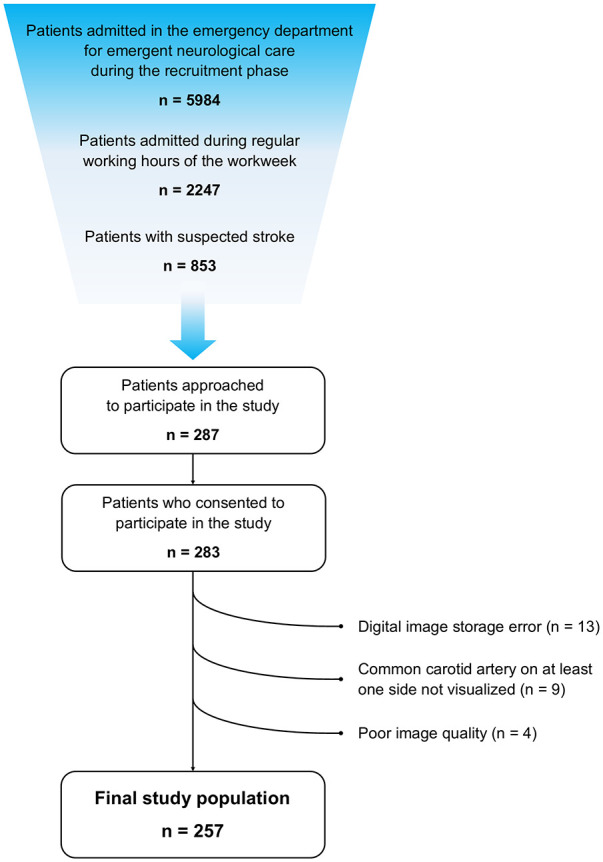
Patient flow diagram.

During the training phase, there was a significant improvement in feasibility and duration of POCUS examination throughout time (Supplemental Table 2). During the recruitment phase of the study 5984 patients were admitted in the ED for emergent neurological care, of whom 2247 patients were admitted during the workweek between 8:00am and 04:00pm. Among these, 853 patients presented with suspected stroke, 287 patients were approached to participate in the study. Only 4 patients refused to participate and 283 patients consented to participate and were examined. Twenty-six patients (9%) were excluded from the final study population: digital image storage error (*n* = 13), non-visualization of CCA on at least one side (*n* = 9), poor image quality (*n* = 4) (Figure 2). The final study population consisted of 257 patients with a median age of 75 years (IQR = 62–83), 131 were female (51.0%), median baseline NIHSS was 2 (IQR = 0–5). The median duration of examinations was 3 min (IQR = 2–5). Detailed baseline characterization of the final study population is presented in Table 1. The most frequent final diagnosis was ischemic stroke (*n* = 127, 49.4%) followed by transient ischemic attack or amaurosis fugax (*n* = 36, 14.0%). ac-LVO was found in 30 patients (11.7%), no patient had an occlusion of the CCA. No patient suffered an adverse event related to the performance of POCUS.

**Table 1.** Baseline characteristics of the final study population.

Baseline characteristics	Study population (*n* = 257)
Age (years)	75 (62–83)
Female sex	131(51.0)
Vascular risk factors and comorbidities	
Arterial hypertension	173 (67.3)
Diabetes	59 (23.0)
Dyslipidemia	78 (30.4)
Current smoking	29 (11.3)
Atrial fibrillation	64 (24.9)
Coronary heart disease	53 (20.6)
Previous stroke	48 (18.7)
Baseline National Institutes of Health Stroke Scale	2 (0–5)
Baseline systolic blood pressure (mmHg)	145 (130–164)
Baseline diastolic blood pressure (mmHg)	83 (73–93)
Door-to-POCUS (minutes)	19 (10–55)
Duration of POCUS examination in minutes	3 (2–5)
Final diagnosis	
Ischemic stroke	127 (49.4)
Transient ischemic attack	36 (14.0)
Peripheral vertigo	11 (4.3)
Sepsis-associated encephalopathy	11 (4.3)
Intracerebral, subdural or subarachnoidal hemorrhage	10 (3.9)
Functional neurological symptoms	6 (2.3)
Non-convulsive status epilepticus or post-ictal focal neurological deficits	5 (2.0)
Other diagnoses	51 (19.8)

POCUS: Point-of-care ultrasound.

Data is presented as *n* (%) or median (interquartile range).

Table 2 presents the characteristics of patients with and without ac-LVO. Patients with ac-LVO more frequently had coronary heart disease (36.7% vs 18.5%, *p* = 0.021), presented higher baseline NIHSS scores (median 16 vs 1, *p* < 0.001), more often received intravenous thrombolysis (30.0% vs 8.4%, *p* < 0.001) and mechanical thrombectomy (76.7% vs 5.7%, *p* < 0.001). Time interval between hospital admission and POCUS was shorter for patients with ac-LVO compared with patients without ac-LVO (median 9 vs 20 min), but this difference was not statistically significant (*p* = 0.057). Duration of examinations was longer for patients with ac-LVO (median 4 vs 3 min, *p* = 0.035). Interobserver agreement for visual diagnosis of ac-LVO was good (κ = 0.71). The distribution of all of the CCA flow wave parameters was significantly different in both groups, with patients with ac-LVO presenting lower PSV and EDV and higher RI and PI on the symptomatic side in comparison with patients with no ac-LVO, and presenting lower PSV and EDV and higher RI and PI on the symptomatic side in comparison with the contralateral side (Table 2).

**Table 2.** Baseline characteristics of the final study population according to the presence of cervical and/or intracranial segments of the internal carotid artery and/or M1 occlusion (ac-LVO).

Baseline characteristics	No ac-LVO (*n* = 227)	ac-LVO (*n* = 30)	*p*
Age (years)	74 (62–83)	79 (72–83)	0.101
Female sex	113 (49.8)	18 (60)	0.293
Vascular risk factors and comorbidities			
Arterial hypertension	149 (65.6)	24 (80)	0.115
Diabetes mellitus	53 (23.3)	6 (20.0)	0.682
Dyslipidemia	66 (29.1)	12 (40.0)	0.221
Current smoking	25 (11.0)	4 (13.3)	0.706
Atrial fibrillation	54 (23.8)	10 (33.3)	0.256
Coronary heart disease	42 (18.5)	11 (36.7)	0.021
Previous stroke	41 (18.1)	7 (23.3)	0.486
Baseline National Institutes of Health Stroke Scale	1 (0–4)	16 (9–19)	<0.001
Baseline systolic blood pressure (mmHg)	145 (129–164)	144 (133–170)	0.916
Baseline diastolic blood pressure (mmHg)	83 (73–94)	80 (68–92)	0.389
Door–to-POCUS (minutes)	20 (12–56)	9 (7–42)	0.057
Duration of POCUS examination (minutes)	3 (2–4)	4 (2–11)	0.035
Ischemic stroke	97 (42.7)	30 (100)	<0.001
Vessel occlusion			<0.001
Internal carotid artery and / or M1	0	30 (100)	
M2 / M3	12 (5.3)	0	
P1 / P2	5 (2.2)	0	
Basilar artery	3 (1.3)	0	
Other vessel (*n*, %)	3 (1.3)	0	
Intravenous thrombolysis	19 (8.4)	9 (30.0)	<0.001
Mechanical thrombectomy	13 (5.7)	23 (76.7)	<0.001
Common carotid artery flow wave parameters			
Peak systolic velocity (cm/s)^a^	42 (27–55)	31 (20–44)	0.012
End-diastolic velocity (cm/s)^a^	9 (6–12)	4 (3–6)	<0.001
Resistance index^a^	0.79 (0.74–0.84)	0.85 (0.82–0.91)	<0.001
Pulsatility index^a^	1.65 (1.44–1.89)	1.97 (1.83–2.35)	<0.001
Peak systolic velocity difference (cm/s)^b^	1 (−9 to 8)	–13 (−23 to −3)	<0.001
End-diastolic velocity difference (cm/s)^b^	0 (−2 to 3)	–7 (−11 to −4)	<0.001
Resistance index difference^b^	–0.01 (–0.05 to 0.03)	0.10 (0.05 to 0.16)	<0.001
Pulsatility index difference^b^	–0.02 (–0.22 to 0.13)	0.51 (0.20 to 0.80)	<0.001

ac-LVO: anterior circulation large vessel occlusion. POCUS: Point-of-care ultrasound.

Data is presented as n (%) or median (interquartile range).

a Symptomatic side.

b Symptomatic side - asymptomatic side.

### Diagnostic performance of POCUS for detection of ac-LVO

Table 3 presents the diagnostic performances of POCUS parameters. The best diagnostic accuracy for detection of ac-LVO was found for EDV difference between sides (AUC = 0.90, 95%CI = 0.85–0.93). At a sensitivity threshold of 80% for continuous POCUS variables, EDV difference between sides had the highest specificity (83%, 95%CI = 78%–88%), the highest PPV (39%, 95%CI = 31%–47%), the highest NPV (97%, 95%CI = 94%–98%) and the highest PLR (4.8, 95%CI = 3.4–6.7). Visual diagnosis of ac-LVO had the highest PLR among all POCUS parameters (13.2, 95% = 6.0–28.8) and presented a high specificity (96%, 95%CI = 93–99). However, it was associated with a low sensitivity (47%, 95%CI = 28%–66%). All of the POCUS parameters presented low PPVs, ranging between 15% and 63%.

**Table 3.** Diagnostic performance of point-of-care ultrasound of the common carotid arteries for the detection of anterior circulation large vessel occlusion (ac-LVO).

Parameters of point-of-care ultrasound	AUC (95%CI)	Sensitivity, % (95%CI)	Specificity, % (95%CI)	PPV, % (95%CI)	NPV, % (95%CI)	PLR (95%CI)
**Symptomatic common carotid artery**						
**Peak systolic velocity**^a^	0.64 (0.58–0.70)	80 (61–92)	42 (36–49)	15 (12–18)	94 (89–97)	1.4 (1.1–1.7)
**End-diastolic velocity**^b^	0.84 (0.79–0.88)	80 (61–92)	70 (63–76)	26 (21–31)	96 (93–98)	2.6 (2.0–3.4)
**Resistance index**^c^	0.80 (0.75–0.85)	80 (61–92)	67 (60–73)	24 (20–29)	96 (92–98)	2.4 (1.9–3.1)
**Pulsatility index**^d^	0.80 (0.75–0.85)	80 (61–92)	67 (60–73)	24 (20–29)	96 (92–98)	2.4 (1.9–3.1)
**Side comparison**						
**Peak systolic velocity difference**^e^	0.70 (0.64–0.75)	80 (61–92)	53 (46–60)	18 (15–22)	95 (91–98)	1.7 (1.4–2.1)
**End-diastolic velocity difference**^f^	0.90 (0.85–0.93)	80 (61–92)	83 (78–88)	39 (31–47)	97 (94–98)	4.8 (3.4–6.7)
**Resistance index difference**^g^	0.87 (0.83–0.91)	80 (61–92)	79 (73–74)	32 (26–39)	97 (93–98)	3.8 (2.8–5.2)
**Pulsatility index difference**^h^	0.88 (0.83–0.92)	80 (61–92)	78 (72–83)	32 (26–39)	97 (93–98)	3.6 (2.7–4.8)
**Visual diagnosis**	0.72 (0.66–0.77)	47 (28–66)	96 (93–99)	63 (45–79)	93 (91–95)	13.2 (6.0–28.8)

AUC: area under the curve; PPV: positive predictive value; NPV: negative predictive value; PLR: positive likelihood ratio; 95%CI: 95% confidence interval.

Individual cut-off values indicative of ac-LVO: ^a^<44 cm/s; ^b^<6 cm/s; ^c^>0.82; ^d^> 1.81; ^e^<0 cm/s; ^f^<−2 cm/s; ^g^>0.03; ^h^>0.13.

The diagnostic performances of the different POCUS parameters for the detection of ICA, M1, M2 and basilar artery occlusions were overall poorer in comparison with detection of only ICA and M1 occlusions (Supplemental Table 3). Despite this, diagnostic accuracy of EDV difference between sides was high (AUC 0.83, 95%CI = 0.78–0.87), with a specificity of 70% at a predefined sensitivity threshold of 80%.

In a second sensitivity analysis, the main results did not differ significantly when analyzing only patients with ischemic stroke as the final diagnosis (Supplemental Table 4). EDV difference between sides was also found to have the best diagnostic accuracy for detection of ac-LVO (AUC = 0.90, 95%CI = 0.83–0.95).

We found 44 patients with false positive POCUS findings according to the chosen cut-off value of EDV difference between sides (<−2 cm/s). In comparison to patients with true negative findings (no ac-LVO and EDV difference between sides ⩾−2 cm/s, *n* = 183), these patients had more frequently a 50%–99% internal carotid artery stenosis ipsilateral to suspected ischemic stroke (11.4% vs 1.1%, *p* = 0.003).

## Discussion

The major finding of this study is that a focused POCUS of the CCAs performed by trained non-specialist personnel accurately predicts the presence of ac-LVO in an unselected population of patients with suspected stroke. Among all the examined parameters, EDV difference between symptomatic CCA and asymptomatic CCA presented the highest diagnostic accuracy.

There is increasing evidence supporting the need for optimization of prehospital processes in patients with ischemic stroke and intracerebral hemorrhage.^14–16^ Screening tools which accurately predict the presence of LVO in patients with suspected stroke may be used in the prehospital setting to select patients which could benefit from a direct transfer to a MT-capable center.^8^ This “mothership” strategy could shorten the time to MT and be associated with better outcomes. We envision a prehospital scenario were continuously trained emergency medical personnel or paramedics use POCUS of the CCAs in patients with suspected stroke, and an automatic analysis of flow wave profiles informs the decision for transport to a nearby hospital or to a MT-capable center.

Requirements of prehospital LVO screening include the need not to miss patients with LVO, to avoid direct transfers to MT-capable centers of patients who do not benefit or are harmed by this strategy and the method should be easily and rapidly performed by prehospital personnel.^17^ POCUS of CCAs showed a high accuracy, acceptable specificity at a predefined threshold of 80% for sensitivity, high NPP for the detection of ac-LVO and it was performed rapidly by non-specialist personnel, thus fulfilling these requirements. The best performing parameter was increased EDV difference between sides (depicting absent or greatly decreased end-diastolic flow in the symptomatic CCA compared with the asymptomatic CCA), and increased PI and RI difference between sides (depicting higher arterial pulsatility and resistance in the symptomatic CCA compared with the asymptomatic CCA). These waveform characteristics are known indirect markers of distal occlusion.^18^ The lower accuracy of visual diagnosis for detection of ac-LVO, supports the use of objective observer-independent cut-off values for POCUS parameters. Overall, low PPV and PLR were found for POCUS parameters. Although low PPV can be partially explained by low pre-test probability, these suboptimal values represent an important limitation of this screening method, which in the prehospital setting could induce increased direct transfers of patients without ac-LVO to thrombectomy centers.

We recognize that a relevant stenosis distal to the examined segment may be a confounding factor. Specifically, a 50%–99% internal carotid artery stenosis may induce waveform changes which can mimic CCA flow wave patterns compatible with ac-LVO, thus representing a limitation of the method.

Prehospital clinical scales have been developed as tools for accurate prediction of LVO and were implemented in stroke code systems.^19^ The PRESTO study analyzed the prehospital performance of eight scales in a population of >1000 patients with suspected stroke.^9^ The scale with the highest accuracy in estimating the likelihood of ICA-, M1- and M2-occlusions was the Rapid Arterial oCclusion Evaluation (RACE),^20^ with an AUC = 0.83, but had a sensitivity of only 67%. Higher sensitivities were found in other studies, in which RACE was performed by experienced neurologists or derived from NIHSS.^21,22^ In the RACECAT trial, one of the reasons which could have contributed to the lack of benefit of directly transferring patients to MT-capable centers in comparison to transporting patients to a local stroke center could have been the suboptimal diagnostic accuracy of RACE.^23^ In our study, we could not compare the screening performance of POCUS with that of clinical scales because they are not routinely performed by prehospital services in our region.

Alternative screening methods such as transcranial Doppler ultrasonography^24^ and electroencephalography^25^ were reported to present excellent screening performance for identification of LVO (defined differently in different studies, but encompassing mostly M1 and M2 occlusions), but have relevant limitations such as insufficiency or lack of temporal bone window in up to 56% of patients and insufficient quality of electroencephalography data in 32% of patients, respectively.

The main limitations of our study include the relatively small population size, the small number of patients with medium vessel occlusions and intracranial hemorrhage which precludes a meaningful analysis of whether CCA flow wave pattern is also influenced by these conditions, and the lack of information on prehospital clinical scales which does not allow a head-to-head comparison of screening methods. POCUS of the CCAs is not expected to identify occlusions of the vertebrobasilar territory or medium vessel occlusions which still may benefit from MT. The duration of the POCUS examinations was relatively short in our study (median of 3 min), but in cases where rapid and sufficient visualization of the flow wave profiles is not possible, the exam needs to be interrupted not to delay diagnostic and treatment procedures. Averaging flow wave metrics throughout multiple heart cycles was not performed in our study and could improve validity of the measurements.

Our results must be interpreted with caution, because the finding of an excellent screening method for LVO does not directly imply that the “mothership” strategy based on this method will induce better patient outcomes. Prehospital stroke code systems are region-specific. The benefit of the mothership strategy greatly depends on the ability of the system to avoid relevant delays of intravenous thrombolysis and to avoid transport delay for patients without ischemic stroke who would otherwise benefit from a faster admission in a nearby hospital.

The main strengths of our study include the number of patients with ac-LVO (12% of the sample), the fact that POCUS was performed by unexperienced examiners who received a structured training (suggesting that transfer of this competence to prehospital personnel may be feasible), the fact that visualization of both CCA with good image quality was possible in 95% of the study population, and the fact that all patients received an emergent CT-angiography or MR-angiography to confirm the diagnosis of ac-LVO.

In conclusion, our study shows that POCUS of both CCAs in patients with suspected acute stroke is feasible and may accurately predict the presence of anterior circulation large vessel occlusion. Further studies analysing in the prehospital phase are needed to confirm these results and address potential limitations related to prehospital personnel training, image quality and image interpretation during transport.

## Supplemental Material

sj-docx-1-eso-10.1177_23969873251315337 – Supplemental material for Point-of-care ultrasound of the common carotid arteries for detection of large vessel occlusion stroke: Results of the POCUS-LVO study Supplemental material, sj-docx-1-eso-10.1177_23969873251315337 for Point-of-care ultrasound of the common carotid arteries for detection of large vessel occlusion stroke: Results of the POCUS-LVO study by João Pinho, Anna Tyurina, Celina Hartmann, Omar Abu Audeh, Pardes Habib, Ramy Abdelnaby, Oliver Matz, Marc Felzen, Jörg C. Brokmann, Martin Wiesmann, Jörg B. Schulz, Omid Nikoubashman and Arno Reich in European Stroke Journal
