# Supplementary material for: Point-of-care ultrasound of the common carotid arteries for detection of large vessel occlusion stroke: Results of the POCUS-LVO study
Source: Eur Stroke J. 2025 Jan 30;10(3):853–61. doi: 10.1177/23969873251315337 (PMC11783414; doi:10.1177/23969873251315337)
Supplement: sj-docx-1-eso-10.1177_23969873251315337 – Supplemental material for Point-of-care ultrasound of the common carotid arteries for detection of large vessel occlusion stroke: Results of the POCUS-LVO study [file sj-docx-1-eso-10.1177_23969873251315337.docx]

SUPPLEMENTARY MATERIAL

Supplementary Table 1: The Standards for Reporting of Diagnostic Accuracy (STARD) Checklist

| **Section and topic** | **Item** | **Description** | **Page** |
| --- | --- | --- | --- |
| Title, abstract and keywords | 1 | Identify the article as a study of diagnostic accuracy (recommended MeSH heading “sensitivity and specificity”) | 2-3 |
| Introduction | 2 | State the research questions or aims, such as estimating diagnostic accuracy or comparing accuracy between test or across participant groups | 5 |
| Methods: |  |  |  |
| Participants | 3 | Describe the study population: the inclusion and exclusion criteria and the settings and locations where the data were collected | 5-6 |
|  | 4 | Describe participant recruitment: was this based on presenting symptoms, results from previous tests, or the fact that the participants had received the index tests or the reference standard? | 5-6 |
|  | 5 | Describe participant sampling: was this a consecutive series of participants defined by selection criteria in items 3 and 4? If not, specify how participants were further selected | 5-6 |
|  | 6 | Describe data collection: was data collection planned before the index tests and reference standard were performed (prospective study) or after (retrospective study)? | 6-7 |
| Test methods | 7 | Describe the reference standard and its rationale | 7 |
|  | 8 | Describe technical specifications of material and methods involved, including how and when measurements were taken, or cite references for index tests or reference standard, or both | 5-7 |
|  | 9 | Describe definition of and ration for the units, cut-off-points, or categories of the results of the index tests and the reference standard | 8 |
|  | 10 | Describe the number, training, and expertise of the persons executing and reading the index tests and the reference standard | 5, 7 |
|  | 11 | Were the readers of the index tests and the reference standard blind (masked) to the results of the other test? Describe any other clinical information available to the readers | 6-8 |
| Statistical methods | 12 | Describe methods for calculating or comparing measures of diagnostic accuracy and the statistical methods used to quantify uncertainty (e.g. 95% confidence intervals) | 8-9 |
|  | 13 | Describe methods for calculating test reproducibility, if done | Not done |

(cont.)

| **Section and topic** | **Item** | **Description** | **Page** |
| --- | --- | --- | --- |
| Results: |  |  |  |
| Participants | 14 | Report when the study was done, including beginning and ending dates of recruitment | 5 |
|  | 15 | Report clinical and demographic characteristics (e.g. age, sex, spectrum of presenting symptoms, comorbidity, current treatments, and recruitment centre) | 10, Table 1 |
|  | 16 | Report how many participants satisfying the criteria for inclusion did or did not undergo the index tests or the reference standard, or both; describe why participants failed to receive either a test (a flow diagram is strongly recommended) | 9-10, Figure 2 |
| Test results | 17 | Report time interval from index tests to reference standard, and any treatment administered between | 6-7 |
|  | 18 | Report distribution of severity of disease (define criteria) in those with the target condition and other diagnoses in participants without the target condition | 10, Table 2 |
|  | 19 | Report a cross tabulation of the results of the index tests (including indeterminate and missing results) by the results of the reference standard; for continuous results, report the distribution of the test results by the results of the reference standard | Table 2 |
|  | 20 | Report any adverse events from performing the index tests or the reference standard | 10 |
| Estimates | 21 | Report estimates of diagnostic accuracy and measures of statistical uncertainty (e.g. 95% confidence intervals) | 11, Table 3 |
|  | 22 | Report how indeterminate results, missing responses and outliers of index tests were handled | NA |
|  | 23 | Report estimates of diagnostic accuracy and measures of statistical uncertainty (e.g. 95% confidence intervals) | 9 |
|  | 24 | Report estimates of test reproducibility, if done | Not done |
| Discussion | 25 | Discuss the clinical applicability of the study findings | 12-13 |

Supplementary Table 2. Feasibility and total duration of point-of-care ultrasound for both common carotid arteries categorized according to the timeline of the training phase (tertile 1 corresponds to the first 48 patients examined, tertile 2 correspond to patients 49-99, tertile 3 correspond to patients 100-150).

|  | Tertile 1  (exams 1-48) | Tertile 2  (exams 49-99) | Tertile 3  (exams 100-150) | p |
| --- | --- | --- | --- | --- |
| Feasibility (n, %)* | 28 (58%) | 42 (82%) | 47 (91%) | <0.001^†^ |
| Total duration in minutes (median, interquartile range) | 7 (5-9) | 4 (4-5) | 3 (2-4) | <0.001^‡^ |

^*^ Feasibility of the point-of-care ultrasound was defined as the presence of images showing both common carotid arteries in the longitudinal plan, correct positioning of the Doppler cursor for visualization of flow wave profiles, visualization of at least three consecutive flow waves of the common carotid artery.

^†^ Chi square test

^‡^ Kruskal Wallis test

Supplementary Table 3. Diagnostic performance of point-of-care ultrasound of the common carotid arteries for the detection of ICA, M1, M2 and basilar artery occlusion.

|  | **AUC**  **(95%CI)** | **Sensitivity, %**  **(95%CI)** | **Specificity, %**  **(95%CI)** | **PPV, %**  **(95%CI)** | **NPV, %**  **(95%CI)** | **PLR**  **(95%CI)** |
| --- | --- | --- | --- | --- | --- | --- |
| **Symptomatic common carotid artery** |  |  |  |  |  |  |
| **Peak systolic velocity^a^** | 0.61 (0.55-0.68) | 80 (65-91) | 39 (32-46) | 20 (17-23) | 91 (84-95) | 1.3 (1.1-1.6) |
| **End-diastolic velocity^b^** | 0.71 (0.65-0.76) | 85 (71-94) | 45 (38-52) | 23 (20-26) | 94 (88-97) | 1.6 (1.3-1.9) |
| **Resistance index^c^** | 0.65 (0.59-0.71) | 80 (65-91) | 38 (31-44) | 20 (17-23) | 91 (84-95) | 1.3 (1.1-1.6) |
| **Pulsatility index^d^** | 0.65 (0.59-0.71) | 80 (65-91) | 38 (31-44) | 20 (17-23) | 91 (84-95) | 1.3 (1.1-1.5) |
| **Side comparison** |  |  |  |  |  |  |
| **Peak systolic velocity difference^e^** | 0.66 (0.59-0.71) | 80 (65-91) | 22 (16-28) | 16 (14-19) | 85 (75-92) | 1.0 (0.9-1.2) |
| **End-diastolic velocity difference^f^** | 0.83 (0.78-0.87) | 80 (65-91) | 70 (64-76) | 34 (28-40) | 95 (91-97) | 2.7 (2.1-3.5) |
| **Resistance index difference^g^** | 0.79 (0.74-0.84) | 80 (65-91) | 66 (60-73) | 31 (26-36) | 95 (91-97) | 2.4 (1.9-3.0) |
| **Pulsatility index difference^h^** | 0.80 (0.74-0.84) | 80 (65-91) | 67 (60-73) | 31 (26-36) | 95 (91-97) | 2.5 (1.9-3.1) |
| **Visual diagnosis** | 0.70 (0.63-0.75) | 41 (26.58) | 96 (93-98) | 68 (50-82) | 90 (87-92) | 11.2 (5.2-24.2) |

AUC: area under the curve. PPV: positive predictive value. NPV: negative predictive value. PLR: positive likelihood ratio. 95%CI: 95% confidence interval.

Individual cut-off values indicative of ac-LVO: ^a^< 47 cm/s; ^b^< 10 cm/s; ^c^>0.76 ; ^d^> 1.56; ^e^< 9 cm/s ; ^f^< -1 cm/s; ^g^> 0.01 ; ^h^> 0.07.

Supplementary Table 4. Diagnostic performance of point-of-care ultrasound of the common carotid arteries for the detection of anterior circulation large vessel occlusion (ac-LVO) only in patients with confirmed ischemic stroke (n=127).

|  | **AUC**  **(95%CI)** | **Sensitivity, %**  **(95%CI)** | **Specificity, %**  **(95%CI)** | **PPV, %**  **(95%CI)** | **NPV, %**  **(95%CI)** | **PLR**  **(95%CI)** |
| --- | --- | --- | --- | --- | --- | --- |
| **Symptomatic common carotid artery** |  |  |  |  |  |  |
| **Peak systolic velocity^a^** | 0.63 (0.54-0.72) | 63 (44-80) | 61 (50-71) | 33 (25-42) | 84 (76-89) | 1.6 (1.1-2.3) |
| **End-diastolic velocity^b^** | 0.84 (0.77-0.90) | 77 (58-90) | 80 (71-88) | 54 (43-65) | 92 (85-96) | 3.9 (2.5-6.1) |
| **Resistance index^c^** | 0.79 (0.71-0.86) | 87 (69-96) | 65 (55-74) | 43 (36-51) | 94 (86-98) | 2.5 (1.8-3.4) |
| **Pulsatility index^d^** | 0.79 (0.71-0.86) | 87 (69-96) | 65 (55-74) | 43 (36-51) | 94 (86-98) | 2.5 (1.8-3.4) |
| **Side comparison** |  |  |  |  |  |  |
| **Peak systolic velocity difference^e^** | 0.69 (0.60-0.77) | 77 (58-90) | 66 (56-75) | 41 (33-50) | 90 (83-95) | 2.3 (1.6-3.2) |
| **End-diastolic velocity difference^f^** | 0.90 (0.83-0.95) | 87 (69-96) | 81 (72-87) | 59 (48-69) | 95 (89-98) | 4.7 (3.0-7.3) |
| **Resistance index difference^g^** | 0.87 (0.79-0.92) | 77 (58-90) | 83 (73-89) | 58 (46-69) | 92 (86-96) | 4.4 (2.7-7.0) |
| **Pulsatility index difference^h^** | 0.88 (0.81-0.93) | 73 (54-88) | 87 (78-93) | 63 (50-75) | 91 (85-95) | 5.5 (3.2-9.5) |
| **Visual diagnosis** | 0.73 (0.64-0.80) | 47 (28-66) | 99 (94-100) | 94 (66-99) | 86 (81-89) | 44.8 (6.1-326.7) |

AUC: area under the curve. PPV: positive predictive value. NPV: negative predictive value. PLR: positive likelihood ratio. 95%CI: 95% confidence interval.

Individual cut-off values indicative of ac-LVO: ^a^< 36 cm/s; ^b^< 5.6 cm/s; ^c^>0.81 ; ^d^> 1.77; ^e^< -4 cm/s ; ^f^< -3 cm/s; ^g^> 0.05 ; ^h^> 0.27.
